# Antibacterial Hydrogels Derived from Poly(γ-glutamic acid) Nanofibers

**DOI:** 10.3390/gels8020120

**Published:** 2022-02-14

**Authors:** Hamidreza Kasbiyan, Omid Yousefzade, Estelle Simiand, Núria Saperas, Luis J. del Valle, Jordi Puiggalí

**Affiliations:** 1 Departament d’Enginyeria Química, Escola d’Enginyeria de Barcelona Est-EEBE, c/Eduard Maristany 10-14, Universitat Politècnica de Catalunya, 08019 Barcelona, Spain; hamidreza.rkh@gmail.com (H.K.); o.yousefzade@gmail.com (O.Y.); estelle.simiand@gmail.com (E.S.); 2 Barcelona Research Center for Multiscale Science and Engineering, Escola d’Enginyeria de Barcelona Est-EEBE, c/Eduard Maristany 10-14, Universitat Politècnica de Catalunya, 08019 Barcelona, Spain

**Keywords:** hydrogels, electrospinning, nanofibers, antibacterial properties, drug release, bacteriophages

## Abstract

Biocompatible hydrogels with antibacterial properties derived from γ-polyglutamic acid (γ-PGA) were prepared from bulk and electrospun nanofibers. The antibacterial drugs loaded in these hydrogels were triclosan (TCS), chlorhexidine (CHX) and polyhexamethylene biguanide (PHMB); furthermore, bacteriophages were loaded as an alternative antibacterial agent. Continuous and regular γ-PGA nanofibers were successfully obtained by the electrospinning of trifluoroacetic acid solutions in a narrow polymer concentration range and restricted parameter values of flow rate, voltage and needle-collector distance. Hydrogels were successfully obtained by using cystamine as a crosslinking agent following previous published procedures. A closed pore structure was characteristic of bulk hydrogels, whereas an open but structurally consistent structure was found in the electrospun hydrogels. In this case, the morphology of the electrospun nanofibers was drastically modified after the crosslinking reaction, increasing their diameter and surface roughness according to the amount of the added crosslinker. The release of TCS, CHX, PHMB and bacteriophages was evaluated for the different samples, being results dependent on the hydrophobicity of the selected medium and the percentage of the added cystamine. A high efficiency of hydrogels to load bacteriophages and preserve their bactericide activity was demonstrated too.

## 1. Introduction

Development of new materials with bactericidal activity involves continuous research that is fundamental for sectors such as healthcare, reparative medicine and even food packaging [1,2,3,4]. The adhesion and proliferation of microorganisms on material surfaces leads to biofilms with organized bacterial microcolonies, which can cause severe health problems, especially in hospital environments [5,6]. Natural agents like bacteriophages and different clinical agents have been considered to prevent infections. Efforts have mainly been focused to incorporate the appropriate biologically active agent into a polymeric matrix. In this sense, a good compatibility between selected polymers and agents, and a high surface-to-mass ratio of the polymeric matrix are desirable in order to avoid unfavourable agent aggregation [7] and to enhance the contact between the agent and the target bacteria [8].

Hydrogels, which can absorb a large amount of water ranging from 10% to thousands of times their own volume, can be obtained by crosslinking a hydrophilic polymer [9,10]. Herein, poly(γ-glutamic acid) (γ-PGA), a polyamino acid of microbial origin, has been chosen as an organic component. This polymer is produced from D- and L-glutamic acid units by several bacterial strains of *Bacillus subtilis* that give rise to different stereochemistry and even different polymer molecular weights. γ-PGA is biocompatible, enzymatically degradable, non-immunogenic and has reactive carboxylic acid (–COOH) side groups, which appear ideal for both functionalization and hydrogel preparation [11,12,13,14].

Applications of polymer-based hydrogels in medicine are extensive (e.g., drug delivery, tissue engineering, wound dressings), cosmetic and other industrial applications (e.g., contact lenses, hygiene products) [15,16,17]. The physical properties, including swelling, permeation, mechanical strength, and surface characteristics can be modulated through structural modifications [18]. Different studies have been performed to prepare hydrogels based on γ-PGA from different crosslinking agents, but probably cystamine, a disulfide-crosslinking agent, is the more effective [19,20]. Published results indicated that the structure and content of the selected agent determined the pore size, the crosslinking degree, the water absorption and even the degradation profile [12,13,21,22]. 

The problems indicated above concerning the encapsulation of active agents into polymeric systems can be minimized by means of the electrospinning technique. This is an easy process that can lead to highly porous scaffolds with a tunable porosity. Furthermore, the constitutive nanofibers can be easily loaded with the selected agent, using different strategies [23,24]. Electrospun nanofibers of hydrophilic polymers can subsequently be crosslinked giving rise to new hydrogels. These hydrogels based on nanofibers of specific diameter are interesting for advanced requirements in the field of biomedical applications. These mainly involve drug delivery systems and regenerative medicine as demonstrated by the increasing number of patents concerning the use of electrospun hydrogels, which can easily offer antimicrobial, anti-inflammatory and antioxidant activities [25]. Furthermore, great efforts are nowadays focused on the scale-up of electrospinning from the laboratory to an industrial production level, with the nanofiber worldwide market size was estimated to noticeably increase up to 1 billion USD by the end of 2020 [26]. 

The use of hydrogels based on γ-PGA is receiving great attention and different attempts have been proposed such as the bonding of the carboxyl group to nanoparticles [27] and the development of ionic hydrogels based on gelatin and γ-PGA [28]. Tajima and Sukigara [29] worked on the mechanical and antibacterial properties of non-woven γ-PGA using oxazoline as a crosslinking agent. These 3D networks showed a swelling-controlled drug release. Wang et al. [30] investigated on the processing conditions required to prepare uniform γ-PGA nanofibers with a smooth morphology, which were then crosslinked using cystamine to amidate the carboxyl groups of γ-PGA. A polymer concentration of 16 wt-%, aqueous solvent containing 1–10 wt-% of 1,1,1,3,3,3-trifluoroacetic acid (TFA), flow rate of 0.3 mL·h^−1^, applied voltage of 32 kV, collector distance of 40 cm, and ambient humidity less than 50% were considered the most appropriate conditions. Furthermore, the disulphide bonds provided by the cystamine crosslinking agent were appropriate for having a reduction-responsiveness and being easily decomposed under physiological conditions using non-toxic reductants such as L-cysteine [19]. 

Triclosan (TCS), chlorhexidine (CHX) and polyhexamethylene biguanide (PHMB) are usually employed as biocides for disinfection in both home and hospital settings. These compounds therefore have been selected in this work as examples of hydrophobic (TCS) and hydrophilic (CHX, PHMB) compounds, as well as low (CHX) and moderate (PHMB) molecular weight compounds. TCS basically acts as an inhibitor of the lipid biosynthesis [31], while CHX and PHMB are characterized by the presence of easily protonated secondary amines (biguanide groups) which are highly effective against microorganisms [32]. 

Phage therapy is the application of bacteria-specific viruses to treat pathogenic bacterial infections. It appears as a highly interesting and cost-effective antibiotic alternative to treat bacterial infections with antibiotic resistance [33,34,35]. Scaffolds based on electrospun hydrophilic fibers have been assayed for the incorporation of bacteriophages [36,37,38] with not completely satisfactory results mainly as a consequence of activity loss caused by the electrospinning process [39]. An alternative and interesting procedure is the use of hydrogels derived from electrospun nanofibers to load without damage the bacteriophages by a simple immersion on the commercial mother liquor. Hydrogels allow the direct delivery of phages to the site of infection and have applications as for example wound dressings, treatment of chronic wounds and treatment of multidrug resistant infections. 

The goals of the present work can be summarized as follows: (a) Preparation of antibacterial hydrogels based on γ-PGA and displaying different morphologies, (b) Evaluation of release characteristics and bactericide activity according to the morphology of the hydrogel (i.e., from bulk or from electrospun nanofibers), crosslinking degree, and specific properties of loaded drugs (i.e., hydrophilicity and molecular weight), (c) Evaluation of the capacity to incorporate bacteriophages through measurements of bactericide activity. 

## 2. Results and Discussion

### 2.1. Electrospinning of γ-PGA

The main problem hindering the electrospinning process was the scarce polymer solubility in the usual organic solvents. Therefore, all assays were carried out in TFA as previously reported [19], since it was the only one of the assayed potential solvents that was able to disrupt the strong hydrogen bonding intermolecular interactions. Another important limitation to use this system for electrospinning was the high viscosity of the polymer solution, which limited the polymer concentration to a value of 16 wt-%. In order to decrease the viscosity, dilutions of TFA with water were also assayed according to previous data [30], but unsuccessful results were attained due to a phase separation in diluted and concentrated polymer solutions.

It is also important to point out the necessity to get a perfect and homogeneous polymer solution. To achieve this, it was essential to extend the solubilization process for a minimum of 72 h. Lower times led to fibers with high size dispersion and even the presence of not completely dissolved particles.

**Figure 1:**
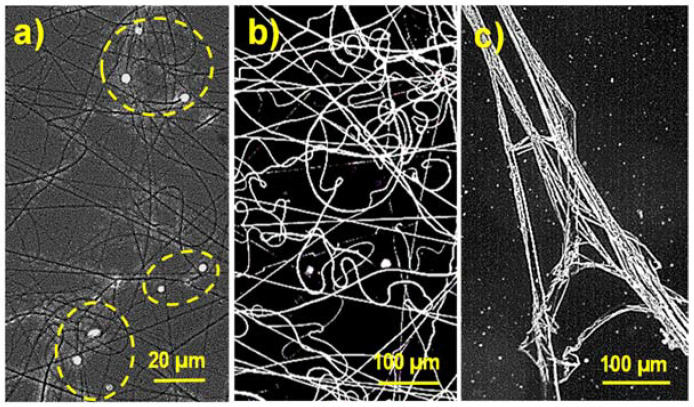
Optical micrographs comparing the morphology of γ-PGA electrospun fibers obtained at concentrations of 3 (**a**), 8 (**b**) and 13 wt-% (**c**) using a voltage of 30 kV, a flow rate of 1 mL/h and a needle to collector distance of 25 cm.

Electrospinning was therefore performed from polymer concentrations varying from 3 to 13 wt-% (i.e., 3, 5, 8, 11 and 13 wt-% concentrations were considered). Formation of a high ratio of droplets and solvent retention were the detected problems for the minimum and maximum concentrations (Figure 1). In general, the best results were achieved for the concentration of 8 wt-%.

A flow rate of 0.5 mL/h was the higher value that allowed getting continuous fibers without an appreciable formation of droplets. The increase of polymer concentration required a decrease on the flow rate. For example, for concentrations > 10 wt-%, flow rate had to be decreased to 0.3 mL/h to get optimal fiber morphology. Concerning the needle tip to collector distance a value of 25 cm was necessary to guarantee a complete evaporation of the solvent and form uniform fibers. The relatively high viscosity needed a high applied voltage (30 kV) to form the Taylor cone and stretch the polymer in the ejected jet.

**Figure 2:**
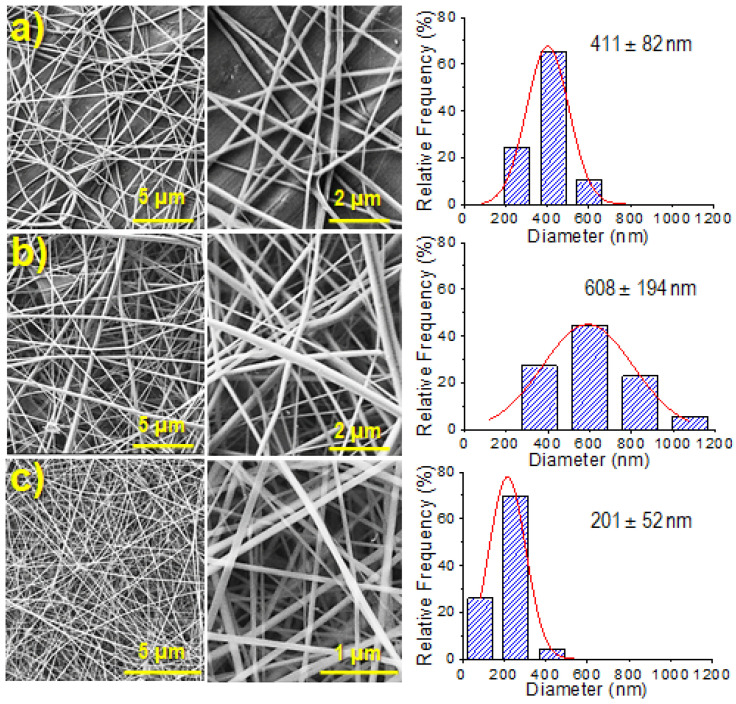
SEM micrographs (low and high magnification) and fiber diameter distribution of γ-PGA electrospun fibers obtained at concentrations of 5 (**a**), 8 (**b**) and 11 wt-% (**c**) using a voltage of 30 kV, a flow rate of 0.5 mL/h (**a**,**b**) or 0.3 mL/h (**c**) and a needle collector distance of 25 cm.

SEM micrographs comparing the fiber morphology achieved under the optimized conditions for three polymer concentrations (5, 8 and 11 wt-%) are shown in Figure 2. Fiber diameter distribution for each concentration is also shown in Figure 2.

It is clear that the number of collected fibers per surface unit increased with polymer concentration as well as the average diameter (see low magnification SEM images of Figure 2a,b). Note that at the higher concentration, the flow rate should be decreased from 0.5 mL/h to 0.3 mL/h, a feature that affected the fiber morphology and that specifically caused a decrease on the average diameter as shown in Figure 2c, i.e., from 411–608 nm to 201 nm. However, the density of deposited fibers was increased for the electrospinning of the higher concentrated solution despite the flow rate was decreased. Note also in Figure 2 that, in all cases, continuous and regular nanofibers with a smooth surface were obtained.

Fiber electrospun mats were highly hydrophilic, being impossible to determine the contact angle of water drops. Evidences of partial solubility were even detected in some cases (preparations with thinner fibers), showing that intermolecular interactions changed from the commercial powder to the electrospun nanofibers.

### 2.2. Preparation of γ-PGA Hydrogels

**Figure 3:**
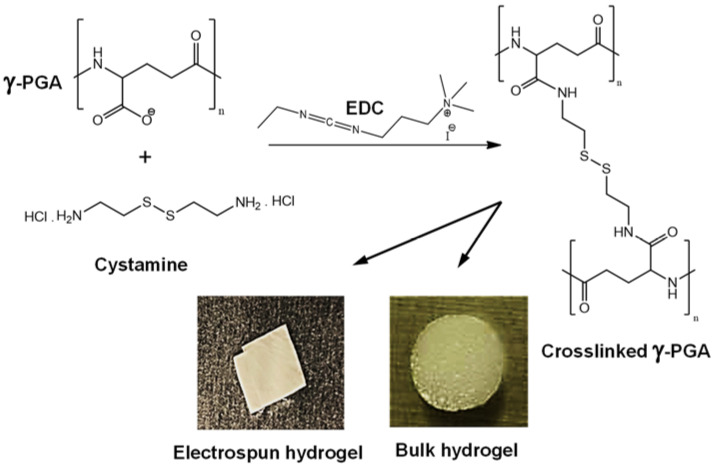
Scheme showing hydrogel preparation from γ-PGA and representative images of the two types of prepared hydrogels.

γ-PGA was crosslinked with cystamine, a molecule having di-terminated amine groups and –S–S– disulfide bonds, by a condensation reaction promoted by EDC as condensing agent. Different types of hydrogels (i.e., bulk or from electrospun mats) were prepared as shown in Figure 3. Crosslinking degree was also changed through the cystamine/polymer ratio used in the condensation reaction.

Bulk hydrogels were easily prepared following the procedure described below. However, this procedure was more complex for the hydrogels obtained from electrospun fibers due to a slight fiber solubilization detected when highly hydrophilic media such as methanol were employed. Therefore, previously reported methods [15,30] were slightly modified and cystamine was specifically dissolved in a methanol/ethanol 35:65 mixture where the polymer was completely insoluble. Furthermore, 3D specimens were prepared by stacking different electrospun mats and sealing the borders of the final set in order to minimize any nanofiber lost during the immersion and washing processes. Reaction time with the cystamine solution was crucial to ensure that the desired crosslinking degree was attained and that the fiber stacking was preserved after repeating washing steps. Thus, assays at different exposure times were performed (i.e., 1, 3, 6 and 24 h), being determined that a minimum reaction time of 6 h was necessary.

**Figure 4:**
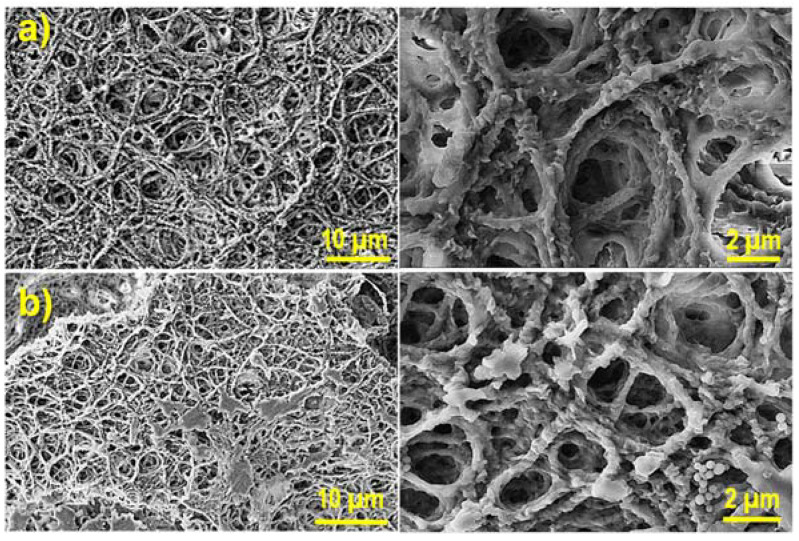
SEM micrographs (low and high magnifications) comparing the morphology of 100% crosslinked hydrogels derived from electrospinning solutions with polymer concentrations of 5 wt-% (**a**) and 8 wt-% (**b**).

Fiber morphology and specially the number of fibers per surface area in the electrospun mat played a relevant role in the morphology of the derived hydrogel. Figure 4 illustrates the differences found between hydrogels with the maximum theoretical crosslinking degree that were prepared from electrospun mats obtained from 5 and 8 wt-% polymer solutions. SEM micrographs revealed despite the logical changes derived from the drying process that a more compact structure was logically observed for the higher concentration of 8 wt-%, which was specifically selected for the following experiments.

### 2.3. Physical Characterization of Hydrogels

**Figure 5:**
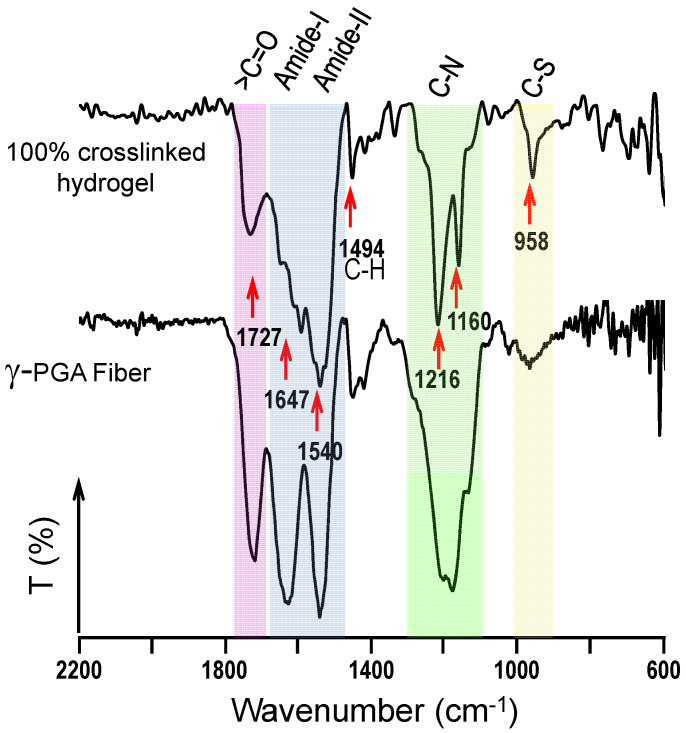
FTIR spectra of a theoretically 100% crosslinked hydrogel obtained from electrospun nanofibers. For comparison, the spectrum of electrospun γ-PGA nanofibers taken before the crosslinking process is also provided. Note that the 100% crosslinking reaction was not complete (see text for further details).

FTIR spectroscopy was carried out to demonstrate that the crosslinking reaction took place and confirm the indirect evidence derived from the physical integrity of the pieces. A representative FTIR spectrum of a 100% crosslinked hydrogel is shown in Figure 5. The strong peaks at 1647 cm^−1^ and 1540 cm^−1^ are assigned to the amide I and amide II bands, respectively, which correspond to amide groups of both the main chain and the crosslinks. It is highly significant that a carboxylic group peak around 1727 cm^−1^ could still be observed (although with a relatively lower intensity) in the hydrogel as presumable from an incomplete amidation. This could be consequence of a stoichiometric default of cystamine caused by the inaccuracy of the polymer molecular weight (i.e., it could be lower than the 350 Kg/mol value used in the theoretical calculations) or even for a partial reaction of cystamine in such a way that is partially incorporated in dangling chains. The small sharp peak at 958 cm^−1^ was also significant in the hydrogel since it corresponds to the introduced C-S bond after the crosslinking process. Note that different relative intensities were found in function of the theoretical crosslinking degree and that the signal around 1647 cm^−1^ can be highly complex.

The enzymatic degradability of γ-PGA has been demonstrated by treatment with hydrolases that break peptide bonds and even are able to produce monomers [40]. However, little information concerns the study of a simple hydrolytic degradation in γ-PGA hydrogels. Weight losses of 0.3% and 4.2% were observed after 18 and 49 days of exposure, demonstrating the chemical stability of hydrogels but also a small susceptibility to the hydrolytic attack. Logically, a reduced weight loss should in any case be expected due to the effective crosslinking between chains. Evidences of hydrolysis could be better detected considering the FTIR data. Nevertheless, infrared spectra of samples exposed for a long time to the degradation medium did not point out an increase of the carboxylic acid band (not shown) as could be expected from the breakage of amide groups. 

**Figure 6:**
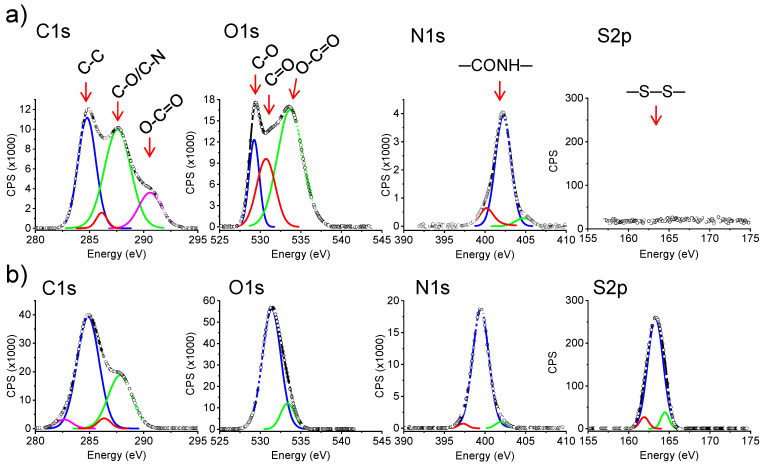
C 1s, O 1s, N 1s and S 2p high resolution XPS spectra for γ-PGA electrospun fibers (**a**) and hydrogel from electrospun fibers crosslinked with the theoretical stoichiometric amount of cystamine (**b**). Deconvolution of the spectra is also shown to indicate the presence of different types of atoms.

XPS spectroscopy was used to further confirm that the crosslinking reaction took place and furthermore to perform a more quantitative analysis. Comparison of characteristic XPS spectra in the C 1s, O 1s, N 1s and S 2p regions showed clear differences in the composition before and after the crosslinking process of electrospun fibers since all reagent should be removed after the extensive washing (Table 1). Results pointed out an increase in the C, N and S atomic percentages and a decrease in the oxygen content. All these features agree with the incorporation of cystamine units in the hydrogel. The C 1s high resolution XPS spectrum was complex due to different types of involved carbon atoms, whereas single peaks were mainly detected for the N 1s and S 2p spectra (Figure 6). The S percentage was relatively low and was an evidence that the crosslinking reaction was not complete for the hydrogels derived from the electrospun fibers. Table 1 indicates also that reticulation reaction was more successful for the bulk hydrogels. The obtained results showed interestingly a linear increase of the S % with the expected degree of crosslinking from the reaction conditions (i.e., cystamine and EDC concentrations). Thus, this percentage was reduced practically to 50% (i.e., from 3.9% to 2.1%) when spectra of hydrogels with theoretical crosslinking degrees of 100% and 50% were compared. Note also the consistent increase in the C and N content and the decrease of O as the reticulation increases as a consequence of the incorporation of cystamine moieties and the loss of some oxygen atoms of free carboxylic groups. Note that variation of the atomic composition cannot be merely ascribed to a simple incorporation of non-reacted cystamine due to the extensive washing process. Unfortunately, the analysis cannot discriminate between cystamine acting as effective crosslinker or belonging to dangling chains. The obtained atom percentages suggest that the maximum cystamine conversion was around 40% and 60% for the electrospun and bulk hydrogels, respectively. In conclusion, systems with high consistence (higher for the bulk hydrogel) were achieved from the crosslinking process, although a significant amount of free carboxylic groups remained in the samples as deduced from FTIR spectra. 

The hydrogel preparation method highly affected on the water uptake, being this higher for samples prepared from the electrospun fibers than for the bulk for a given theoretical crosslinking degree. Obviously, water uptake decreased with the increase of the crosslinking density. Porosity, surface tension and the amount of available area for contacting water play an important role to determine the solvent uptake (SU). Experimental values were calculated from the weights of the swollen (W_s_) and lyophilized (W_d_) hydrogels (Equation (1)):

SU (%) = ((W_s_ − W_d_)/W_d_) × 100 (1)

The averaged values over three independent measurements are given in Table 2.

Macroscopic SU values demonstrated that the swelling capacity of hydrogels can be drastically modified according to the preparation method and that those hydrogels coming from electrospun nanofibers may present water uptake values higher by around two-three times (i.e., 170% versus 492%), which may be highly interesting for drug release applications if an enough mechanical consistence of hydrogels can be maintained. Furthermore, water uptake can be easily tuned by modifying the crosslinking degree (i.e., from 332% to 492% when the theoretical crosslinking decreases from 100% to 50%).

### 2.4. Morphology of Hydrogels

**Figure 7:**
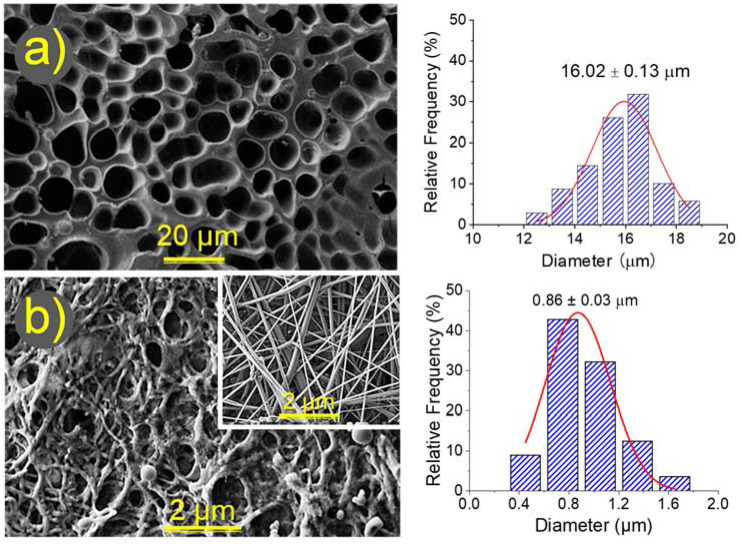
SEM micrographs (**left**) comparing the morphology of hydrogels prepared from the bulk (**a**) and from electrospun nanofibers (**b**) after reaction with the stochiometric ratio of cystamine 100%. In both cases pore diameter distributions are provided (**right**). For comparison purposes, the inset of (**b**) shows the electrospun fibers before performing the crosslinking reaction.

The high difference in the water uptake values than can be found between bulk and electrospun hydrogels can be correlated with the respective morphology, as shown in Figure 7. In this figure, SEM micrographs of both types of dried hydrogels are shown together with the pore diameter distribution.

The average pore diameter found in the bulk hydrogel was 16 µm, a value significantly higher than that determined from the space between fibers for the electrospun hydrogel (0.8 µm). It seems that this factor was not essential because water accessibility to the inner material occurred without any difficulty. Nevertheless, the closed (bulk hydrogel) and the open (hydrogel from electrospun fibers) pore structures played a role as well as the surface/volume ratio of γ-PGA, which was clearly lower for the bulk hydrogel. Figure 7b also reveals the dramatic change in the electrospun fiber morphology after the crosslinking reaction. Thus, irregular and rough surfaces with globular textures could be observed in the hydrogels in contrast with the smooth and uniform initial fibers. SEM micrographs pointed out that, even for the less crosslinked sample, the initial fiber morphology was partially disturbed and, in addition, a noticeable adhesion between fibers was detected. 

**Figure 8:**
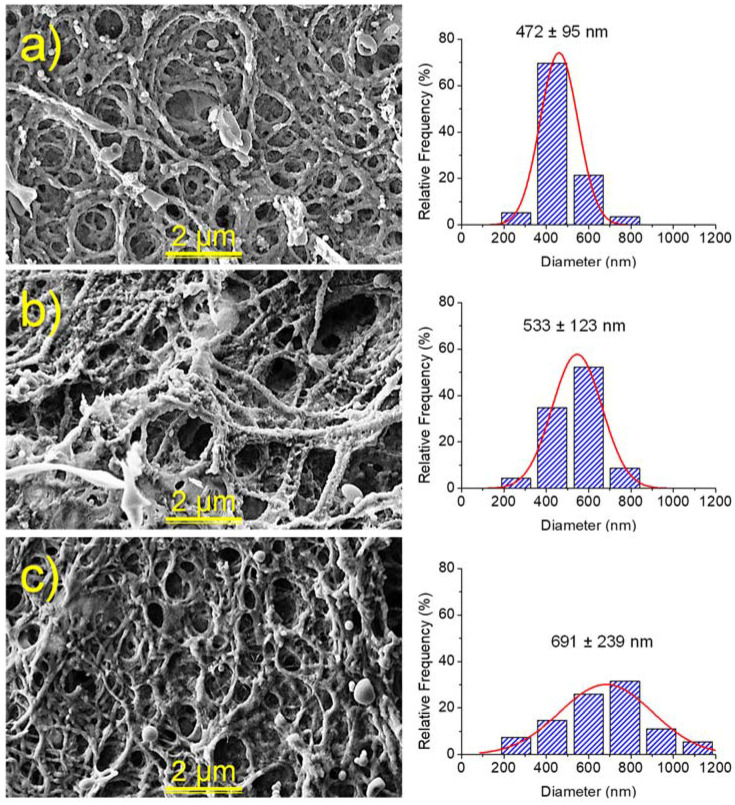
SEM micrographs (**left**) comparing the morphology of hydrogels from electrospun nanofibers and a crosslinking process involving 50% (**a**), 75% (**b**) and 100% (**c**) of the steochiometric amount of cystamine. Fiber diameter distributions are provided in the **right**.

The morphology of hydrogels also changed with the degree of crosslinking. Thus, the pore size decreased in both bulk and electrospun hydrogels. Nevertheless, in the last case the greater influence was on the fiber diameter size, which increased with crosslinking due to the incorporation of an increasing amount of cystamine inside the individual nanofibers (Figure 8). Average diameters of 472, 533 and 691 nm were estimated for samples with crosslinking degrees of 50%, 75% and 100%, respectively.

### 2.5. γ-PGA Hydrogels for the Release of Triclosan as a Hydrophobic Drug

**Figure 9:**
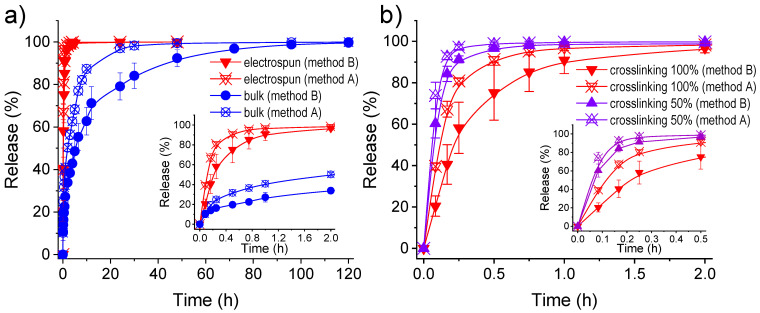
Accelerated release of triclosan from hydrogels in a PBS-ethanol (70v:30v) medium. (**a**) TCS relative release percentages for electrospun and bulk hydrogels; both with a crosslinking degree of 100%. (**b**) TCS relative release percentages for electrospun hydrogels with different crosslinking percentages. In both graphics, methods A and B refer to the loading of TCS by absorption or during the crosslinking process, respectively. Insets show release determined at initial times.

Figure 9a compares triclosan (TCS) release from the bulk and the electrospun hydrogel with a theoretical crosslinking degree of 100%. Furthermore, the behavior is also compared according to the two methods used to load the drug: i) absorption into the hydrogel (method A), and ii) encapsulation into the polymer network during the crosslinking reaction (method B). The amount of loaded drug was higher for the bulk hydrogel than for the electrospun hydrogel, independently of the used method. Thus, retention values (Table 3) of 5.4% versus 1.6% (method A) and 4.5% versus 2.3% (method B) were obtained, respectively. Encapsulation capability (EC) was evaluated according to Equation (2):

EC (%) = (W_l_/W_dh_) × 100 (2)

where W_l_ and W_dh_ correspond to the weight of loaded drugs and the dried loaded hydrogel, respectively.

TCS was poorly released (low and non-quantifiable values) when PBS, which has a hydrophilic character, was used as the release medium. However, when the hydrophobicity of the release medium was increased (e.g., a 7:3 *v*/*v* mixture of PBS and ethanol) [41,42], the TCS release kinetics could be determined. In this case, release was always very fast and complete due to the low affinity of the hydrophobic drug with the hydrophilic pol2ymer matrix. Nevertheless, results clearly indicated a slower release of triclosan from the bulk hydrogel independently of the loading method, which is fully consistent with its lower water uptake capacity and its closed pore structure. Differences in the saturation time were found for the bulk hydrogel according to the loading process (Figure 9a). Specifically, the 48 h saturation time detected when TCS was loaded by absorption could be increased up to 120 h when TCS was encapsulated into the inner parts. The release from electrospun hydrogels extended only up to 1 h (method A) or 2 h (method B) (Figure 9b). An immediate bactericide effect should be expected for such hydrophobic loaded samples, a distinctive behaviour from hydrophilic drugs as then will be discussed. TCS was a useful drug model to differentiate loading procedures and drastic morphologic changes on the hydrogel morphology.

The decrease on the crosslinking degree of the electrospun hydrogel had little influence on a release that is already very fast. Thus, 1 h (method B) and 0.5 h (method A) are required to attain the complete release when the crosslinking degree was reduced to 50%. In any case, a certain capacity to tune the release is obtained through changes of the morphology, the loading method and the crosslinking degree. Release from the different hydrogels was well simulated using the combined kinetic equation (Table 3). This model usually describe the first part (0–60%) with the Higuchi model (Equation (3)) [43] and the second part (40–100%) with the first-order model (Equation (4)) [44]:

(M_t_/M_0_) = k_H_t^1/2^     0 ≤ (M_t_/M_0_) ≤ 0.6 (3)

Ln (1 − (M_t_/M_0_)) = a − k_1_t     0.4 ≤ (M_t_/M_0_) ≤ 1.0 (4)

where, k_H_ and k_1_ are the Higuchi and first-order release constant, respectively. M_t_ is the percentage of drug released at time t, and M_0_ is the drug equilibrium percentage (considered as the maximum drug percentage).

### 2.6. γ-PGA Hydrogels for the Release of Chlorhexidine and Polyguanide as Hydrophilic Drugs

**Figure 10:**
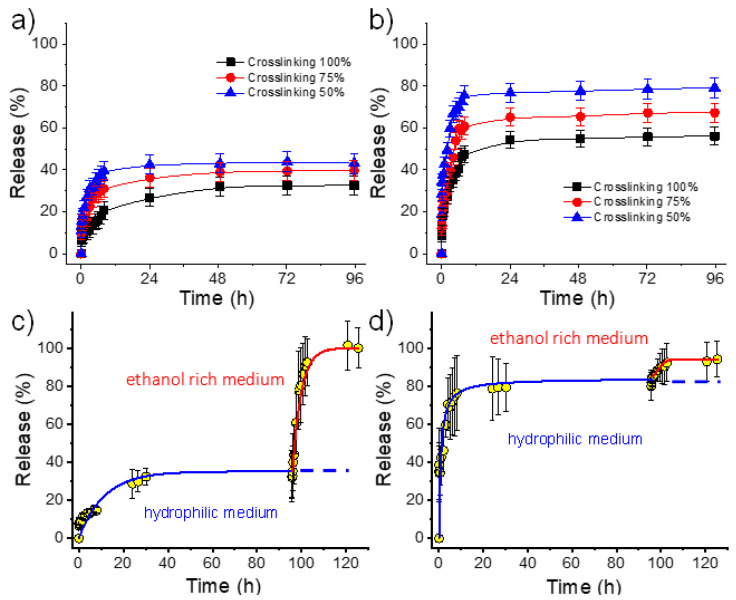
Relative release percentages of CHX (**a**) and PHMB (**b**) from electrospun hydrogels in PBS medium. Two-step release of CHX (**c**) and PHMB (**d**) from electrospun hydrogels with a theoretical crosslinking degree of 50%. The first step was performed in PBS and the second one in the PBS-ethanol medium.

Figure 10 shows the relative release in the PBS medium of CHX and PHMB from electrospun hydrogels having theoretical crosslinking degrees of 100, 75 and 50%. Several points deserve attention:

(1) Both hydrophilic drugs can be released to the hydrophilic PBS medium in contrast with the virtual zero release of the hydrophobic TCS.
(2) PHMB has a greater burst effect than CHX and has also a higher release rate (Table 3, and Figure 10). Differences are highly significant and indicate a clearly higher difficulty of the larger PHMB molecules to be encapsulated into the inner parts of the hydrogel, and even higher affinity with the PBS release medium due to its higher hydrophilicity. In fact, encapsulation capacities (EC, Equation (2)) around 0.07% and 0.78% were determined for CHX and PHMB, respectively, for the 100% crosslinked hydrogels. Note also that highly different saturation levels were reached during the release. Specifically, values of 25% and 52% were found after 96 h of exposure for CHX and PHMB loaded samples with the higher theoretical crosslinking degree, respectively.
(3) In all cases, the release rate was slightly dependent on the theoretical crosslinking degree and logically decreased when it increased (Table 3).
(4) A practically complete release was found for both drugs using a PBS/ethanol mixture since saturation problems could be avoided. In this case, the release rate was found to be slightly higher for CHX. Figure 10c,d clearly demonstrated that the retained drug after PBS exposure could be completely released by a simple change of the medium. Specifically, after 24–96 h of exposure to PBS, the release increased from 40% to 100% for CHX and from 80% to 92% for PHMB when ethanol was added to the medium.

### 2.7. Antibacterial Activity of γ-PGA Hydrogels Loaded with Drugs

**Figure 11:**
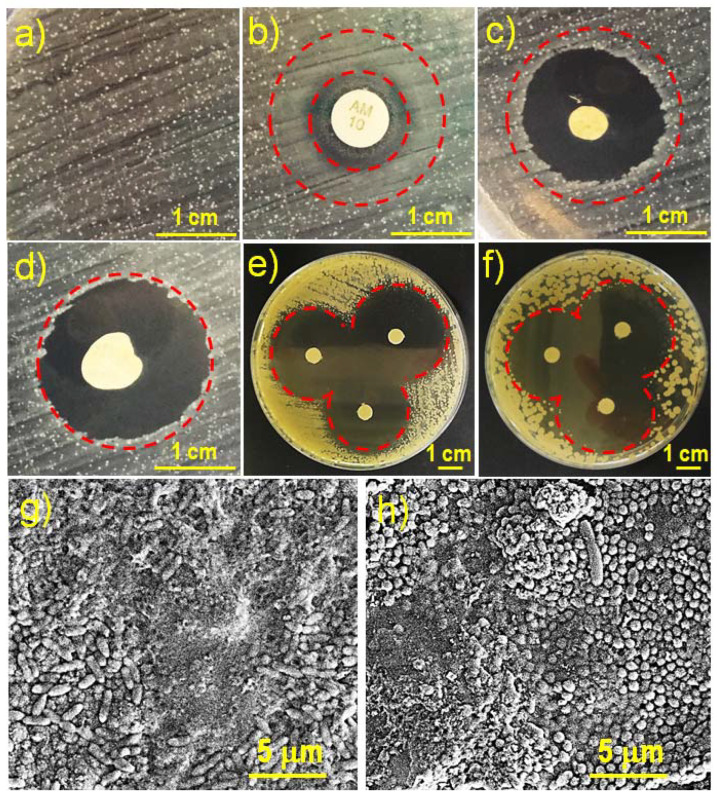
Inhibition of bacterial growth. Growth of the *Staphylococcus aureus* Gram-positive bacteria on a LB agar plate, control (**a**), bactericide effect (inner circle) and bacteriostatic effect (outer circle) of the ampicillin antibiotic (**b**), and bactericide activity of the hydrogels having a theoretical crosslinking degree of 50% and loaded with CHX and (**c**) and PHMB (**d**). For comparison, the outer circle is drawn the same size in (**b**–**d**). Inhibition of antimicrobial solution containing 6.2 *w*/*v*-% of CHX (**e**) and 1.5 *w*/*v*-% of PHMB (**f**). SEM images of the bacteria *E. coli* (**g**) and *S. aureus* (**h**) forming biofilms on the surface of the hydrogel.

The antibacterial activity of the hydrogels loaded with CHX, PHMB and TCS was qualitatively determined by the plate bacterial growth inhibition assay. We have used this typical test to determine the activity of the selected drugs once loaded in the prepared hydrogels against the Gram-positive bacterium *Staphylococcus aureus*. Figure 11 shows the formation of bacterial growth inhibition halos around the hydrogel samples. This halo occurs due to radial diffusion of antibacterial compounds released from hydrogels.

The normal growth of the *S. aureus* bacterium on the agar plate led to bacterial colonies which appear as tiny and small white dots (Figure 11a). As a positive control for antibacterial activity, a disk with an ampicillin load of 10 µg was used (Figure 11b). Ampicillin has a low antibacterial activity against *S. aureus* as demonstrated by the small inhibition halo around the disc that corresponds to the area where no bacterial colony is observed. Then, an area corresponding to a bacteriostatic effect can be observed as revealed by the growth of tiny colonies (Figure 11b). In this sense, the hydrogels loaded with CHX (Figure 11c) and PHMB (Figure 11d) exhibit pronounced bactericide activity, as can be appreciated by the conspicuous halos that are formed around the hydrogel (black area without bacterial colonies). It is noteworthy that the size of both halos (i.e., from CHX and PHMB loaded samples) were similar. This can be justified considering that a CHX load of 6.2% provides an equivalent number of active groups than a PHMB load of 1.5%. This same effect was observed when comparing the inhibitory effect of antibacterial solutions containing 6.2% CHX and 1.5% PHMB (Figure 11e and Figure 11f, respectively). Furthermore, it is important to indicate that the observed bactericidal effect is highly desirable since a simple bacteriostatic effect could lead to eventual bacterial resistance against the selected antimicrobial compounds. Figure 11g,h show SEM images of the bacteria *E. coli* and *S. aureus*, respectively. These samples correspond to hydrogels with a crosslinking degree of 100% and infected for 48 h. The bacteria are clearly observed: *E. coli* with a typical rod morphology and *S. aureus* with a spherical shape. Colonization and biofilm formation with mucilage secretion that closes and collapses the pores and surface of the hydrogel are detected. These observations justify the need to load the hydrogel with antimicrobial agents to avoid bacterial contamination that compromises its function. The antimicrobial-loaded hydrogels prevent bacterial infection and their morphology is similar to that observed in Figure 8.

The antimicrobial activity of the TCS released from the electrospun hydrogels corresponded to a bactericidal effect (data not shown) similar to that observed from the release of CHX and PHMB. These results are logical due to the facility of the selected hydrogels to deliver the selected drugs and to the well-probed effectivity of the drugs against bacteria. Specifically, TCS and CHX are widely used in various personal care products and have been studied as additives in various biomedical materials such as surface disinfection liquids, surgical sutures, and scaffolds for tissue regeneration [41,42,45,46,47]. 

### 2.8. Potential of γ-PGA Hydrogels for an Efficient Load of Bacteriophages

**Figure 12:**
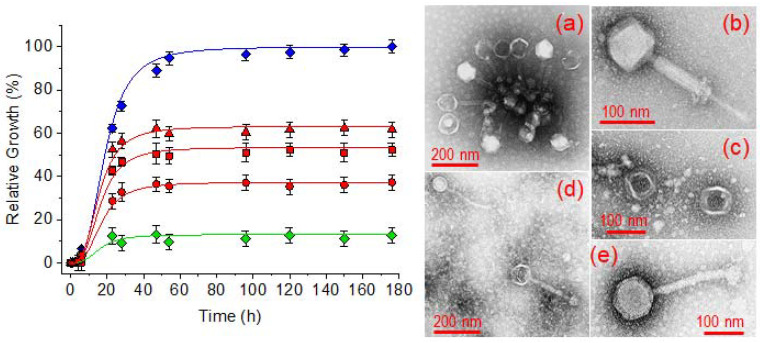
Antibacterial activity of the Fersisi bacteriophages. Control of the *Staphylococcus aureus* bacterial growth (blue), relative growth of *S. aureus* in the commercial preparation of phages (green) and bacteriophage-loaded electrospun hydrogels (red) with crosslinking degrees of 100% (circles), 75% (squares) and 50% (triangles). TEM micrographs show the morphology of Fersisi bacteriophages (commercial preparation): (**a**) *Myoviridae*, (**b**) *Siphoviridae*, (**c**) *Leviviridae*, and (**d**,**e**) *Podoviridae*.

The efficiency of electrospun scaffolds to incorporate bacteriophage preparations has been assayed with a commercial preparation of Fersisi bacteriophages. To this end, a bacterial culture of *Staphylococcus aureus* in a BHI broth containing 10^3^ CFU/mL was used as a control of the maximum bacterial growth. The bacterial growth of *S. aureus* cultures in the presence of phage-loaded hydrogels with different crosslinking degrees was evaluated. Finally, the original Fersisi preparation was used as a positive control of bacterial growth inhibition by lytic activity. Figure 12 shows in all cases a growth characterized by a lag and an exponential growth phase. The lag phase extended over 6 h, after which the exponential growth took place and reached a maximum around 50 h of culture. After that, a stationary phase was observed. A much lower maximum growth was observed for all the cultures grown in the presence of the hydrogels. Thus, a maximum relative growth of 37%, 47%, and 63% was observed for the hydrogels coming from electrospinning and treated with 100%, 75% and 50% of the stoichiometric amount of cystamine, respectively. On the other hand, an 87% of bacterial inhibition was achieved in the presence of the commercial Fersisi preparation. If we consider that the original activity of the Fersisi bacteriophages is 10^10^ PFU/mL, we can establish that the hydrogels progressively released bacteriophages corresponding to 7 × 10^9^, 5 × 10^9^ and 4 × 10^9^ PFU/mL. These values correspond to the release of 70%, 50% and 40% of encapsulated bacteriophages in the hydrogels with 100%, 75% and 50% crosslinking, respectively. Therefore, the bacteriophage release would be related to the water uptake capacity of the hydrogels. Results indicate that hydrogels based on γ-PGA electrospun nanofibers are an appropriate medium to keep the activity of bacteriophages and to provide a biocompatible reservoir to release all bacteriophages in a localized way. It should be pointed out that the studied hydrogels have ionizable carboxylic groups, which could be in conflict with the negative charge of the more voluminous and external part (capsid) of bacteriophages (see bacteriophage morphology in Figure 12) [48]. However, no negative effect was detected, probably due to the slightly acidic pH of the commercial bacteriophage preparation.

### 2.9. Cytotoxicity of γ-PGA Hydrogels

To evaluate the biocompatibility of the γ-PGAs hydrogels, the cytotoxicity assay of the material extract was selected [42]. Hydration of the dry hydrogel caused swelling of the matrix and in turn the exchange of compounds from incomplete synthesis. These should be minimal after the extensive washing process but some traces not detectable in chemical analyses could still be present and cause cytotoxicity. In other cases and depending on the period of time used to prepare the extract, products of early hydrolytic degradation can be recovered. In this sense, the evaluation of an extract of the material is necessary to assess the biocompatibility of the material.

**Figure 13:**
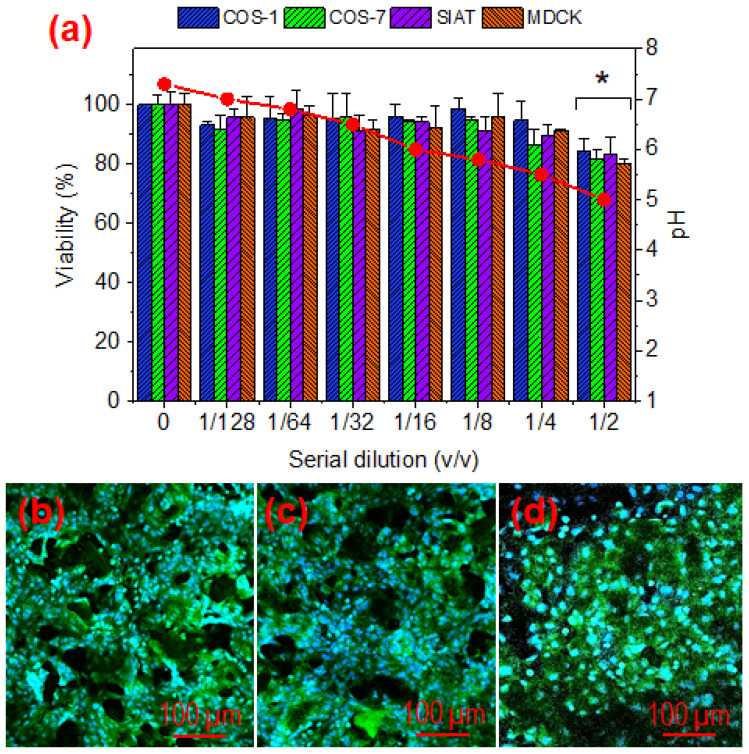
Cytotoxic effect of the γ-PGA hydrogels extract on different cell lines (COS-1, COS-7, SIAT and MDCK). pH (red circles) corresponds to dilution and was not adjusted. The bars are mean ± SD (*n* = 3). * *p* < 0.05, scaffold vs. control, ANOVA followed by Tukey test (**a**). Fluorescence images of MDCK cells cultured for 96 h on hydrogels with 50% (**b**), 75% (**c**), and 100% (**d**) crosslinking degrees. The discontinuity of the monolayer is due to the pores of the hydrogel.

Figure 13 shows the results attained with the cytotoxicity assays performed with hydrogel extracts. Different dilutions of the extracts were evaluated using cell lines of the epithelial (MDCK and MDCK-SIAT) and fibroblast (COS-1 and COS-7) types. Cell viability was perfectly preserved at high dilutions, but a certain cytotoxic damage was detected for both cell lines at a 1:2 dilution. This cytotoxic effect seems to be related with the pH of the extract solution. Thus, the acidic components of the hydrogel led to an extract with pH = 5 that tends to a neutral value as the extract is diluted. As the dilution in the body fluid will be high, no cytotoxic effect is expected after application of hydrogels and therefore they can be considered biocompatible.

Qualitative evidence of cellular biocompatibility on hydrogels has been demonstrated by culturing MDCK cells on hydrogels with different crosslinking degrees. The results (Figure 13b–d) demonstrate the formation of a cell monolayer at 96 h of culture, with discontinuities corresponding to the hydrogel pores. These results clearly demonstrate the biocompatibility of the hydrogel and its physical and mechanical stability for a 50–100% crosslinking degree that seem appropriate to support cell colonization.

## 3. Conclusions

γ-PGA is a biocompatible and hydrophilic polymer suitable for the preparation of antibacterial hydrogels. Carboxylic side groups of the main polyamide chain can be effectively crosslinked by using the disulphide agent cystamine in the presence of a carbodiimide to favour the condensation reaction. γ-PGA can be electrospun, although conditions are highly limited due to its high insolubility in most organic solvents and to its relatively high molecular weight. 1,1,1,3,3,3-trifluoroacetic acid was the most appropriate solvent for electrospinning, being necessary to control the dissolution process which had to be extended for a minimum of 72 h to ensure complete dissolution. Polymer concentration was also primordial to avoid excessive viscosity and droplet formation for high and low values, respectively. An open pore structure was characteristic of electrospun hydrogels, in contrast with the closed pore distribution observed for bulk hydrogels. All hydrogels had a bactericide effect when loaded with the tested drugs (triclosan, chlorhexidine and polyhexamethylene biguanidine). Release rates were clearly dependent on the type of hydrogel, the hydrophilicity of the selected drug, the characteristics of the release medium and, in a minor way, on the crosslinking degree. In summary, different tools are available to tune the bactericide effect of γ-PGA hydrogels. Furthermore, those prepared from electrospinning showed enough mechanical consistency to be locally applied as a fully biocompatible bacteriophage reservoir/dispenser that maintains the activity against *Staphylococcus aureus* bacteria.

## 4. Materials and Methods

### 4.1. Materials

Free-acid γ-PGA was produced by *Bacillus subtilis* isolated from Chungkookjang. The polymer (with *M_w_* average molecular weight between 200 and 500 Kg/mol and D:L ratio of 7:3) was purchased from Wako Chemicals GmbH (Neuss, Germany). TFA was purchased from Merck (Darmstadt, Germany), 2,2-dithiobisethanamine dihydrochloride (cystamine) from Fluka ( Morristown, NJ, USA), 1-[3-(dimethylamino)propyl]-3-ethylcarbodiimide methiodide (EDC, methiodide) and sodium hydrogen bicarbonate from Sigma-Aldrich (St. Louis, MO, USA). All products were used as received.

Triclosan (5-chloro-2-(2,4-dichorophenoxy) phenol) (TCS) and chlorhexidine (1,1′- hexamethylene-bis-5-(4-chlorophenyl) biguanide) (CHX) antibacterial drugs were provided by Sigma-Aldrich. Cosmocil^®^ (polyhexamethylene biguanide hydrochloride, PHMB) was kindly provided by B. BRAUN Surgical S.A. (Barcelona, Spain). 3-(4,5-Dimethylthiazol-2-yl)-2,5-diphenyl- 2H-tetrazolium bromide (MTT) was purchased from Sigma-Aldrich. Cell culture labware and reagents were purchased from Thermo-Fisher Scientific (Barcelona, Catalonia, Spain).

Fersisi bacteriophage preparation was provided by the G. Eliava Institute of Bacteriophages, Microbiology and Virology of Georgia (Tbilisi, Georgia). This preparation is a sterile filtrate of phage lysates of *Staphylococcus* (*S. aureus* and *S. epidermidis*) and *Streptococcus* (*S. pyogenes*, *S. sanguinis*, *S. salivarius* and *S. agalactiae*). Activity of the commercial solution according to the manufacturer was 10^10^ PFU/mL (plate forming units/mL).

Madin-Darby canine kidney (MDCK) cells and MDCK-SIAT cells (derived by the stable transfection of MDCK cells with the cDNA of human 2,6-sialtransferase) were used as epithelial-like cells. COS-1 and COS-7 (from monkey African green kidney) cells were assayed as fibroblast-like cells.

### 4.2. Preparation of γ-PGA Bulk Hydrogels

γ-PGA hydrogels were prepared according to the procedure indicated by Matsusaki et al. [49]. Briefly, γ-PGA (0.5 moles considering a molecular weight of 350 Kg/mol, namely the intermediate value of the interval given by the manufacturer) and the appropriate ratio of EDC (i.e., 0.5 moles) as condensing agent were dissolved in 750 µL of sodium hydrogen carbonate solution (0.5 M) at 4 °C under magnetic stirring. Then, 250 µL of a 0.5 M sodium hydrogen carbonate solution containing 0.25 moles of cystamine were added under stirring for 3 min. The given volume of the reaction solution (1 mL) was poured after removing the magnetic bar into specific containers according to the characterization technique or the application of the sample. Finally, the solution was let to gel at room temperature for three hours. To remove any compound in excess, the resulting hydrogels were extensively washed by soaking in distilled water that was renewed each 12 h for 3 days.

### 4.3. Electrospinning of γ-PGA Nanofibers

For electrospinning, γ-PGA powder was dissolved in TFA (varying the concentration from 3 to 13 wt-%) under magnetic stirring for 72 h. The electrospinning system consisted of a 10 mL syringe having a stainless steel needle with an inner diameter of 0.8 mm, a KDS100 infusion syringe pump (KD Scientific, Holliston, MA, USA) to control the mass-flow rate (from 0.5 to 5 mL·h^−1^), and a high voltage power supply from 10 to 30 kV (ES30-5W, Gamma High Voltage Research, Ormond Beach, FL, USA). A thin aluminum foil (10 cm × 10 cm) was used as a conductive plate, being this collector grounded and horizontally positioned at distances between 15–25 cm from the needle tip. Diameters of electrospun fibers were measured with the SmartTiff software (Carl Zeiss SMT, Ltd., Oberkochen, Germany) from scanning electron microscopy images.

### 4.4. Preparation of Hydrogels from Electrospun γ-PGA Nanofibers

γ-PGA electrospun fibers were collected for 8 h on the aluminum foil. This was then cut into squared 1 cm^2^ pieces, and the fiber scaffolds were peeled off the substrates. Cystamine, as previously indicated, was used to react with the carboxyl groups of γ-PGA through EDC coupling. Briefly, 7–10 layer pieces of γ-PGA nanofibrous mats (1.17 mg) were stacked and then the borders of the set sealed by heating in a Kofler plate. The set was subsequently immersed into an ethanol solution of EDC (740 mg in 1 mL) for 6 h in order to activate the carboxyl groups of γ-PGA [30]. Then, sets were immersed into a cystamine methanol/ethanol 35:65 solution with a concentration of 280 mg/mL for 6 h at 70 °C. The obtained samples were maintained in distilled water for 48 h to remove any remaining compound in the nonwoven fibers. To reduce the hydrogel crosslinking density to theoretical values of 75% or 50%, 555 or 370 mg of EDC and 210 or 140 mg of cystamine were used, respectively.

### 4.5. Characterization

IR absorption spectra for lyophilized γ-PGA hydrogels were recorded on a model 4100 FTIR spectrophotometer (Jasco, Easton, PA, USA). Samples were placed in an attenuated total reflection accessory (top-plate) with a diamond crystal (model MKII Golden Gate Heated Single Reflection Diamond ATR, Specac, Orpington, UK). For each sample, 32 scans were performed between 4000 and 600 cm^−1^ with a resolution of 4 cm^−1^.

The morphology of nanofibers and γ-PGA hydrogels was observed by scanning electron microscopy (SEM) using a Focused Ion Beam Neon 40 SEM (Zeiss) equipped with an energy dispersive X-ray (EDX) spectroscopy system and operating at 5 kV. All samples were sputter-coated with a thin carbon layer using a K950X Turbo Evaporator (Fedelco S.L., Madrid, España) to prevent electron charging problems. Prior to SEM observation, γ-PGA hydrogels were lyophilized (i.e., freeze-dried) and consequently images reflect the secondary morphologies resulting from drying. The size of pores and fibers were determined from the SEM images using the SmartTIFF software, and data was fitted to a normal curve (OrginPro software, OriginLab, Northampton, MA, USA).

X-Ray photoelectron spectroscopy (XPS) analyses were performed in a SPECS system equipped with a high-intensity twin anode X-ray source XR50 of Mg/Al (1253 eV/1487 eV) operating at 150 W, placed perpendicular to the analyzer axis, and using a Phoibos 150 MCD-9 XP detector (SpecsGroup, Berlin, Germany). The X-ray spot size was 650 µm. The pass energy was set to 25 and 0.1 eV for the survey and the narrow scans, respectively. Charge compensation was achieved with a combination of electron and argon ion flood guns. The energy and emission current of the electrons were 4 eV and 0.35 mA, respectively. For the argon gun, the energy and the emission current were 0 eV and 0.1 mA, respectively. The spectra were recorded with a pass energy of 25 eV in 0.1 eV steps at a pressure below 6x10^−9^ mbar. These standard conditions of charge compensation resulted in a negative but perfectly uniform static charge. The C 1s peak was used as an internal reference with a binding energy of 284.8 eV. The surface composition was determined using the manufacturer sensitivity factors.

### 4.6. Hydrolytic Degradation of Hydrogels

Hydrolytic degradation of hydrogels was also studied. To this end 25 mg of the corresponding hydrogel were introduced in 5 mL of a medium (phosphate-buffered saline (PBS) at pH 7.4 and 37 °C) that was renewed each 48 h and sample pieces were extracted at regular time intervals. Samples were then frozen, lyophilized and weighted.

### 4.7. Drug Loading

The highly hydrophobic TCS drug was loaded according to two methods: a) Method A, immersion during 24 h of the selected hydrogel (~25 mg) into a PBS/ethanol (90:10 *v*/*v*) solution containing a TCS concentration of 8 wt-% and subsequent freeze drying. The solvent mixture was selected due to its high capacity to swell γ-PGA nanofibers and keep TCS soluble; b) Method B, incorporation of TCS (the same weight ratio than applied for the nanofiber mats) in the cystamine solution used for the crosslinking process. In this case, a higher encapsulation of TCS is expected initially, although the total load should subsequently decrease due to the washing processes of the hydrogel after reaction.

CHX and PHMB were loaded according to the first method and using solutions containing 6.2 *w*/*v*-% of CHX and 1.5 *w*/*v*-% of PHMB. Note that a lower percentage of PHMB was employed due to its higher ratio of active biguanide groups. Obviously, the second methodology could not be applied due to the hydrophilicity of drugs and the extensive washing.

### 4.8. Drug Release Studies from γ-PGA Hydrogels

Controlled release measurements were performed with 4 mg of loaded hydrogels with a square surface of 0.5 cm × 0.5 cm. Samples were incubated at 37 °C in an orbital shaker at 80 rpm in tubes containing 10 mL of the release medium. Phosphate-buffered saline (PBS) was employed as a release medium, although a 7:3 *v*/*v* mixture of PBS and ethanol was considered for accelerated release studies [41,42]. To determine the drug concentration released, samples (1 mL) were drawn from the release medium at different intervals of time and an equal volume of fresh medium was added to the release container. Drug concentration was evaluated by UV absorbance measurements using linear calibration curves determined for each drug and release medium. A UV–Vis NIR 3600 spectrophotometer (Shimadzu, Kyoto, Japan) was used to carry out the measurements. Spectra were recorded in the absorbance mode at room temperature, the wavelength range and bandwidth being 190–400 nm and 2 nm, respectively. Absorbance measurements were performed at 280, 261 and 236 nm for TCS, CHX and PHMB, respectively.

All drug release tests were carried out using three replicates and the results obtained were averaged. The combination of the Higuchi [43] and first-order [44] models was employed to describe the first (0–60%) and last part of the release (40–100%), respectively.

### 4.9. Bactericidal Activity of the Drugs Loaded into the Electrospun γ-PGA Hydrogels

The antibacterial activity of the hydrogels loaded with TCS, CHX and PHMB was evaluated with a bacterial growth inhibition assay where the formation of the inhibition halos is determined. For this, *Staphylococcus aureus* bacteria were plated by extension on LB agar plates. Then, the hydrogel samples were placed on the agar and the plates were incubated for 24 h. Visualization of inhibition halos was considered as positive antibacterial activity.

### 4.10. Bioactivity of Bacteriophages Loaded in Electrospun γ-PGA Hydrogels

Bacteriophages were loaded by immersion of the hydrogels in 1 mL of the sterile Fersisi bacteriophage solution for 24 h at 37 °C. Then, these hydrogels were dried by lyophilization. *Staphylococcus aureus* bacterium was selected to evaluate the antibacterial effect of bacteriophage-loaded electrospun hydrogels. The bacteria were previously aerobically grown to exponential phase in broth culture (brain heart infusion medium, BHI). Growth experiments were performed in polystyrene tubes using 5 mL of broth. Square pieces (1 cm × 1 cm) of the electrospun hydrogel weighting approximately 5 mg were placed into Eppendorf microtubes. Then, 1 mL of broth culture containing 10^3^ colony forming units (CFU) was seeded on the electrospun fiber mats. The cultures were incubated at 37 °C and agitated at 200 rpm. Aliquots of 50 µL were taken at predetermined time intervals for absorbance measurement at 595 nm in a plate reader. Thus, turbidity was directly related to bacterial growth. All assays were conducted in triplicate and the values averaged.

### 4.11. Cytotoxicity of γ-PGA Hydrogels

The biocompatibility of the hydrogel samples was evaluated in-vitro by a hydrogel extract cytotoxicity test according to the ISO 10993-5 [50] with modifications to prevent the microbial contamination. The hydrogel was extracted in cell culture medium and the extract was then placed in cell containers (96-well tissue culture plate). Cells were examined at 24 h for signs of toxicity. The hydrogel extract was prepared according to the following procedure: the hydrogel samples were incubated in culture medium, in a ratio of 100 mg /mL (mg of hydrogel: mL of medium), for 7 days at 37 °C under sterile conditions (this time is necessary to achieve an exhaustive extraction of the toxic products). For this, sterile 15 mL-tubes were used. After incubation, the tubes were centrifuged at 5000 rpm for 10 min. The culture media were recovered aseptically and filtered using a 0.22 µm syringe filter, and finally stored at −20 °C until evaluation.

MDCK, MDCK-SIAT1, COS-1 and COS-7 cells were cultured in Dulbecco’s modified Eagle’s medium (DMEM with 4500 mg/L of glucose, 110 mg/L of sodium pyruvate and 2 mM of L-glutamine) supplemented with 10% fetal bovine serum (FBS), 50 U/mL penicillin, 50 mg/mL streptomycin and L-glutamine 2 mM at 37 °C in a 10% humidified atmosphere of 5% CO_2_ and 95% air. Culture media were changed every two days. For sub-culture, cell monolayers were rinsed with PBS and detached by incubating them with 0.25% trypsin/EDTA for 2–5 min at 37 °C. The incubation was stopped by resuspending in 5 mL of fresh medium and the cell concentration was determined by counting with a Neubauer camera and using 4% trypan blue as vital dye.

1 × 10^4^ cells were seeded in 96-well plates for 24 h to allow adhesion. Then, the culture medium was removed by aspiration and were replaced by serial dilutions of the hydrogel extract prepared in fresh DMEM. These dilutions were in the range of 1/2 to 1/128 (*v*/*v*). The assay controls consisted of cells not exposed to the extracts. Cultures were maintained for 24 h and then cell viability was determined by the MTT assay [42]. Culture media were aspirated and cells washed twice with PBS, then 100 µL of culture medium supplemented with MTT reagent at a final concentration of 1 mg/mL was added to each well. Plates were incubated for 3 h (during this time viable cells convert the MTT reagent to insoluble formazan salts). The medium was then aspirated and 100 µL of DMSO was added to each well to dissolve the formazan salts and determine viability by measuring absorbance at 570 nm. The experiments were performed in triplicate and the values averaged.

Qualitative evidence of cell growth on the hydrogels was obtained by culturing MDCK cells for 96 h on the hydrogels. The samples were then fixed with 2.5% formaldehyde for 30 min. The samples were then washed 5 times in distilled water, air-dried, and then stained with DAPI and Phalloidin for fluorescent staining of the nucleus and actin of the cytoplasm, respectively.

### 4.12. Statistical Analysis

Frequency or percentage data were averaged and represented as averages ± SD. Statistical analysis was performed with the MiniTab^®^ 18 software (MiniTab, State College, PA, USA) and the graphical representations were performed with the OriginPro v8 software (OriginLab, Northampton, MA, USA). Comparisons between groups were made by ANOVA followed by the Tukey test. The level of significance was considered 5% (*p* < 0.05).

## Figures and Tables

**Table 1:** Atomic percentages deduced from XPS spectra of electrospun hydrogels with different theoretical crosslinking percentages, electrospun mat and bulk hydrogel with a theoretical 100% crosslinking degree.

Sample (%, Theoretical Crosslinking Degree)	C 1s (%)	O 1s (%)	N 1s (%)	S 2p (%)
0% (electrospun mat)	55.5	33.3	11.1	0
50% (hydrogel from electrospun fibers)	56.2	29.2	12.5	2.1
75% (hydrogel from electrospun fibers)	56.0	27.0	13.3	3.3
100% (hydrogel from electrospun fibers)	56.9	25.5	13.7	3.9
100% (bulk hydrogel)	57.4	22.2	14.8	5.0

**Table 2:** Averaged solvent uptake percentage (SU) determined for the different hydrogels.

Sample (%, Theoretical Crosslinking Degree)	SU (%)
50% (hydrogel from electrospun fibers)	492 ± 20
75% (hydrogel from electrospun fibers)	400 ± 17
100% (hydrogel from electrospun fibers)	332 ± 15
100% (bulk hydrogel)	170 ± 10

**Table 3:** Release kinetic parameters for γ-PGA hydrogels loaded with antibacterial drugs. Drugs were loaded by absorption (method A) or during the crosslinking reaction (method B). r, correlation coefficient. EC, encapsulation according to Equation (2).

Hydrogel Sample (%, Crosslinking)	EC	Drug/Loading Method	Release Medium	Higuchi Constant		First Order Constant	
	(%)			k_H_ (h^−0.5^)	r	k_1_ (h^−1^)	r
100% Bulk	5.41	TCS/method A	PBS/EtOH	0.230	0.974	0.042	0.996
100% Electrospun	0.32	TCS/method A	PBS/EtOH	1.024	0.980	1.528	0.980
50% Electrospun	1.59	TCS/method A	PBS/EtOH	1.968	0.993	8.536	0.993
100% Bulk	4.46	TCS/method B	PBS/EtOH	0.336	0.962	0.126	0.992
100% Electrospun	0.46	TCS/method B	PBS/EtOH	1.590	0.994	1.443	0.964
50% Electrospun	2.32	TCS/method B	PBS/EtOH	2.151	0.978	3.932	0.927
100% Electrospun	0.07	CHX/method A	PBS	0.157	0.995	0.064	0.997
75% Electrospun	0.12	CHX/method A	PBS	0.202	0.956	0.080	0.990
50% Electrospun	0.19	CHX/method A	PBS	0.323	0.995	0.238	0.994
100% Electrospun	0.78	PHMB/method A	PBS	0.278	0.988	0.110	0.981
75% Electrospun	2.92	PHMB/method A	PBS	0.294	0.984	0.132	0.956
50% Electrospun	4.39	PHMB/method A	PBS	0.304	0.991	0.307	0.993

## Data Availability

Not applicable.
